# Severe Methemoglobinemia due to Sodium Nitrite Poisoning

**DOI:** 10.1155/2016/9013816

**Published:** 2016-08-03

**Authors:** Kenichi Katabami, Mineji Hayakawa, Satoshi Gando

**Affiliations:** Division of Acute and Critical Care Medicine, Department of Anesthesiology and Critical Care Medicine, Hokkaido University Graduate School of Medicine, Sapporo, Hokkaido 060-8638, Japan

## Abstract

*Case*. We report a case of severe methemoglobinemia due to sodium nitrite poisoning. A 28-year-old man was brought to our emergency department because of transient loss of consciousness and cyanosis. He was immediately intubated and ventilated with 100% oxygen. A blood test revealed a methemoglobin level of 92.5%.* Outcome*. We treated the patient with gastric lavage, activated charcoal, and methylene blue (2 mg/kg) administered intravenously. Soon after receiving methylene blue, his cyanosis resolved and the methemoglobin level began to decrease. After relocation to the intensive care unit, his consciousness improved and he could recall ingesting approximately 15 g sodium nitrite about 1 hour before he was brought to our hospital. The patient was discharged on day 7 without neurologic impairment.* Conclusion*. Severe methemoglobinemia may be fatal. Therefore, accurate diagnosis of methemoglobinemia is very important so that treatment can be started as soon as possible.

## 1. Introduction

Although methemoglobin levels of >70% are generally fatal, patients with methemoglobin levels of up to 94% have survived [1]. Sodium nitrite intoxication is a common cause of severe methemoglobinemia; however, only one suicidal case has been reported [2]. The concentration of methemoglobin does not exceed 1%-2% in the normal physiological state [3] and levels of 10%–20% generally cause cyanosis. On the other hand, methemoglobin levels of 20%–50% may cause symptoms such as respiratory distress, dizziness, headache, and fatigue. Furthermore, loss of consciousness and death can occur at methemoglobin levels of 50%–70% [4]. Methylene blue is the first choice for treating acute methemoglobinemia. It functions along with natural reduction systems to convert methemoglobin to normal hemoglobin. It is typically administered at doses of 1-2 mg/kg body weight intravenously over 5 min, with symptom improvement expected immediately after the administration. In this report, we describe the successful treatment of a case of severe methemoglobinemia due to sodium nitrite poisoning.

## 2. Case Presentation

**Figure 1:**
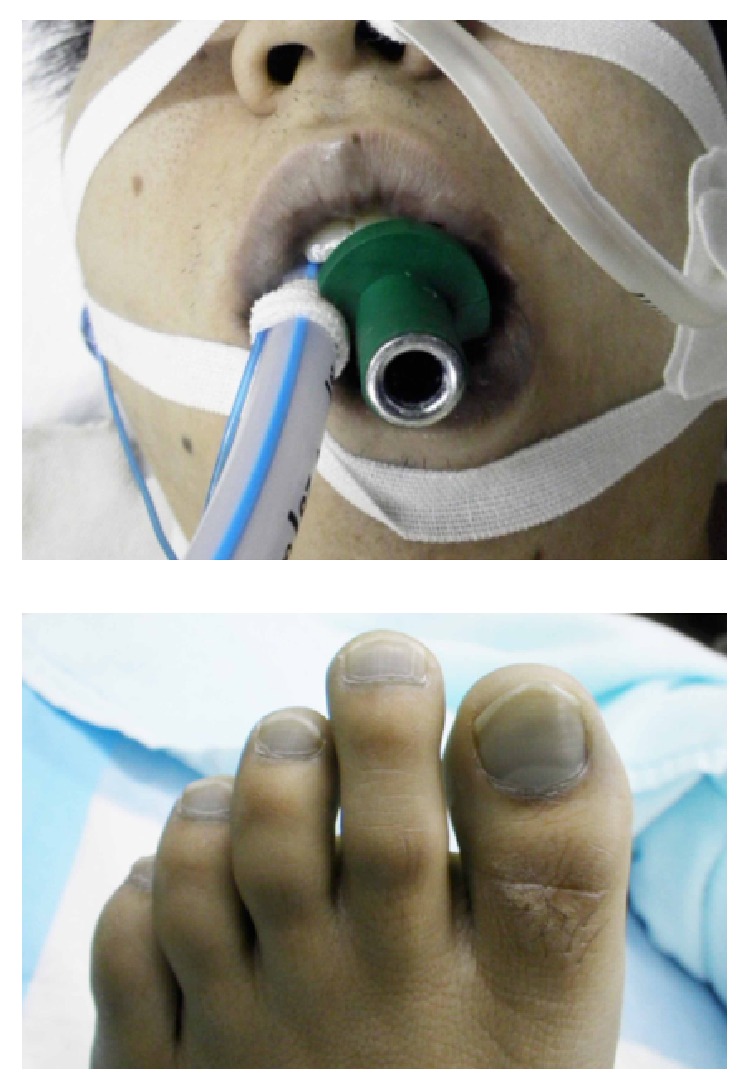
His face, lips, and toes were deeply cyanosed on admission.

A 28-year-old man was brought to our emergency department because of transient loss of consciousness and cyanosis. He was immediately intubated without concomitant drug administration and ventilated with 100% oxygen. Heart rate was 72 beats/min and blood pressure was 82/50 mmHg; oxygen saturation was undetectable on pulse oximetry. His Glasgow Coma Scale score was 3 and he had blue-gray discoloration of the skin, particularly of the face and nail beds (Figure 1).

He was initially treated with 100% oxygen, gastric lavage, and activated charcoal administration. Arterial blood gas analysis and blood tests revealed the following: pH, 7.31; PaCO_2_, 31.4 mmHg; PaO_2_, 564 mmHg; base excess, −10.2 mmol/L; sodium, 137 mmol/L; potassium, 3.2 mmol/L; lactate, 12.1 mmol/L; and methemoglobin, 92.5%. The patient was immediately given 150 mg methylene blue (2 mg/kg body weight) intravenously over 5 min only at one time. He regained consciousness, and cyanosis resolved within minutes after methylene blue injection; methemoglobin concentration decreased to 19% after 60 min.

**Figure 2:**
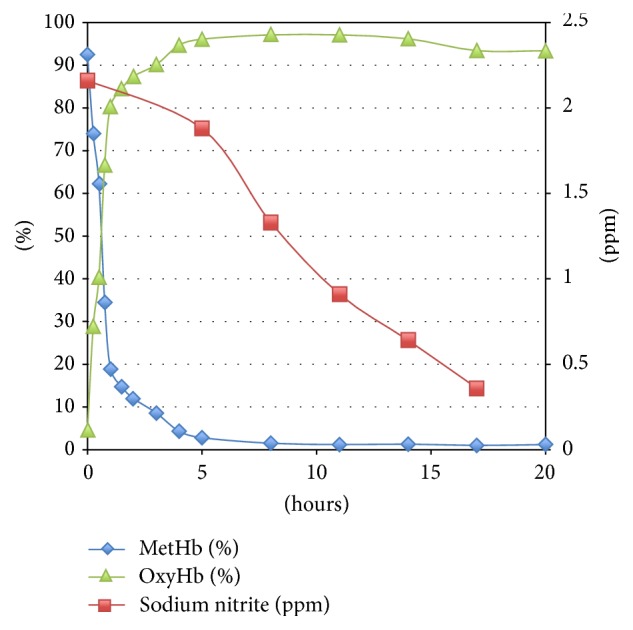
Changes in the serum concentration of sodium nitrite and percentages of methemoglobin and oxyhemoglobin after admission.

**Figure 3:**
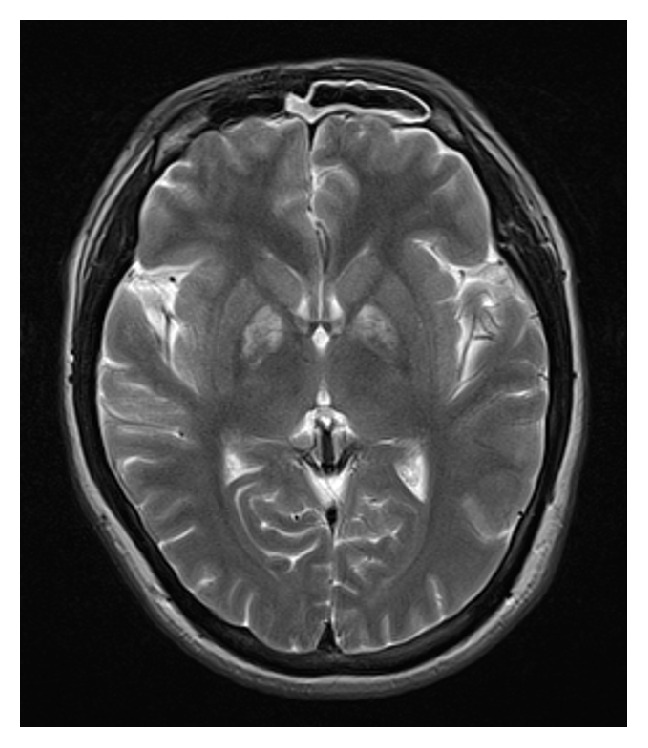
Axial T2-weighted MRI image in a patient with severe methemoglobinemia shows increased signal intensity bilaterally in the globus pallidus.

On the second day of admission, the patient was extubated. He then recalled intentionally ingesting approximately 15 g sodium nitrite about 1 hour before ambulance call. Methemoglobin level was again determined along with serum concentration of sodium nitrite. Methemoglobinemia resolved soon after injection of methylene blue; however, the serum concentration of sodium nitrite decreased gradually (Figure 2). And there was no rebound methemoglobin formation given the persistence of sodium nitrite in the patient. Cranial T2-weighted magnetic resonance imaging (MRI) demonstrated bilateral and symmetrical hyperintense lesions in the globus pallidus (Figure 3). The patient was transferred to the general ward and was subsequently discharged on day 7 without neurologic impairment.

## 3. Discussion

Sodium nitrite is generally used as a coloring agent or preservative in food and as an antimicrobial agent in meat products. The estimated lethal dose of sodium nitrite in adults is approximately 2.6 g [5]; however, a case of a patient surviving after ingesting 6 g sodium nitrite has been reported [6]. Severe methemoglobinemia with fatal outcomes following ingestion of sodium nitrite and intentional self-poisoning have been reported [3, 7].

The initial sign of methemoglobinemia is cyanosis [8] and the diagnosis should be considered in all patients who present with cyanosis, particularly if it does not improve with supplemental oxygen. As the levels reach 30%–40%, symptoms such as headache, fatigue, tachycardia, weakness, and dizziness are experienced. Methemoglobin levels of 60% produce lethargy, convulsions, and coma. Methemoglobin levels of >70% are generally lethal, although survival has been reported with a level of 94% [2]. Nitrite is also a potent vasodilator and can cause coronary ischemia and stroke as a result of hypotension, tachycardia, and hypoxia.

Methylene blue [9] is indicated as the first-line antidote therapy for patients with severe methemoglobinemia. It is recommended that patients with methemoglobin levels >30%, high risk factors such as anemia, or symptoms at any level should be treated using methylene blue at a dose of 1-2 mg/kg body weight intravenously over 5 min. Generally, methemoglobin concentration decreases significantly within 1-2 h after a single dose; additional doses may be necessary. In this case, we could suspect whether the patient had some poisoning because of his cyanosis and severe methemoglobinemia. So we could use methylene blue very quickly in the emergency room.

Cranial T2-weighted MRI findings 3 days after sodium nitrite ingestion were similar to those in carbon monoxide poisoning. It has been reported that the globus pallidus is most susceptible to hypoxia. Severe methemoglobinemia can cause severe tissue hypoxia similar to that in carbon monoxide poisoning; this may explain the involvement of the globus pallidus in our case.

In conclusion, we reported a case of severe methemoglobinemia secondary to intentional ingestion of sodium nitrite. Methemoglobinemia should be considered in all cyanotic patients who are unresponsive to oxygen therapy. Rapid diagnosis and early intervention with methylene blue infusion can prevent a fatal outcome as in the present case with an initial methemoglobin level of 92.5%.
